# Characterization of meningococcal carriage isolates from Greece by whole genome sequencing: Implications for 4CMenB vaccine implementation

**DOI:** 10.1371/journal.pone.0209919

**Published:** 2018-12-28

**Authors:** Konstantinos Kesanopoulos, Holly B. Bratcher, Eva Hong, Athanasia Xirogianni, Anastasia Papandreou, Muhamed-Kheir Taha, Martin C. J. Maiden, Georgina Tzanakaki

**Affiliations:** 1 National Meningitis Reference Laboratory (NMRL), Dept of Public Health, National School of Public Health, Athens, Greece; 2 Department of Zoology, Peter Medawar Building, University of Oxford, Oxford, United Kingdom; 3 Institute Pasteur, Invasive Bacterial Infections Unit, Paris, France; Universidad Nacional de la Plata, ARGENTINA

## Abstract

Herd protection, resulting from the interruption of transmission and asymptomatic carriage, is an important element of the effectiveness of vaccines against the meningococcus. Whilst this has been well established for conjugate polysaccharide vaccines directed against the meningococcal capsule, two uncertainties surround the potential herd protection provided by the novel protein-based vaccines that are used in place of serogroup B (MenB) polysaccharide vaccines (i) the strain coverage of such vaccines against carried meningococci, which are highly diverse; and (ii) the generation of a protective immune response in the mucosa. These considerations are essential for realistic estimates of cost-effectiveness of new MenB vaccines. Here the first of these questions is addressed by the whole genome sequence (WGS) analysis of meningococci isolated from healthy military recruits and university students in Greece. The study included a total of 71 MenB isolates obtained from 1420 oropharyngeal single swab samples collected from military recruits and university students on voluntary basis, aged 18–26 years. In addition to WGS analysis to identify genetic lineage and vaccine antigen genes, including the Bexsero Antigen Sequence Type (BAST), the isolates were examined with the serological Meningococcal antigen Typing System (MATS) assay. Comparison of these data demonstrated that the carried meningococcal population was highly diverse with 38% of the carriage isolates showed expression of antigens matching those included in the 4CMenB vaccine. Our data may suggest a limited potential herd immunity to be expected and be driven by an impact on a subset of carriage isolates.

## Introduction

*Neisseria meningitidis*, the meningococcus, despite its propensity to cause invasive meningococcal disease (IMD) principally meningitis and septicaemia worldwide, is an obligate commensal of the human nasopharynx, found asymptomatically colonising approximately 10% of the human population [1,2]. Although the processes whereby asymptomatic carriage develops into invasive disease remains incompletely understood, genetic analysis of carriage and disease isolates shows that the diversity of carried meningococci is higher, with cases of invasive disease predominately caused by a relatively few genotypes, known as hyperinvasive lineages. These are recognised by multi-locus sequence typing (MLST) analyses as particular clonal complexes (cc) [1,3]. Colonisation is a prerequisite to disease and requires the bacteria to adhere to the mucosal surface, exploit locally available nutrients, and evade human immune reponses. Multiple meningococcal factors facilitate colonization, including pilus-medited attachment to the epithelial cell surfaces and the opacity-associated adhesins [4].

The immunochemical structure of the *N*. *meningitidis* capsular polysaccharide defines 12 serogroups, but only six of these (serogroups A, B, C, W, Y and to lesser extent X) are responsible for most IMD [5]. For example, in the early 21^st^ century in Europe, MenB accounted for 74% IMD cases, MenC for 16%, and MenY and MenW for 5% and 3% respectively [6]. Meningococcal capsule synthesis is encoded by the capsule operon region of the genome and particular capsules tend to be associated with given clonal complexes (ccs). Virtually all IMD isolates are capsulate, although very rare cases of IMD caused by non-capsulate meningococci occur worldwide and these organisms lack the *cps* region, the so-called ‘capsule null’ (*cnl*) meningococci [7]. By contrast, many meningococcal carriage isolates are categorized as non-groupable (NG), by virtue of not expressing a capsule, and can be *cnl*, have a down-regulated capsule synthesis, or be genetically damaged in the *cps* region [8,9]. For instance, in a carriage study in Italy [10], the majority of NG isolates (72.7%) were *cnl*, with other NG isolates containing down-regulated serogroup B capsule synthesis genes. Highly effective polysaccharide conjugate vaccines are available to control groups A, C, W, and Y IMD. Studies during meningococcal serogroup C conjugate (MCC) vaccine implementation demonstrated sustained protection for both vaccinated and non-vaccinated populations, showing both direct and herd protection induced by MCC vaccines [11,12].

The monovalent conjugate vaccine for MenC was introduced in Greece on January 2001 and included in the national immunization program in 2005 in older children and adolescents, with estimated vaccination coverage from 20.7% (2001) to 51.4% (2005) Since April 2011, the quadrivalent meningococcal conjugate vaccine (MCV4) was included in the national immunization programme as a booster dose in adolescents 11–16 years old [13]

The 4CMenB vaccine, a recombinant multicomponent vaccine (Bexsero, GSK), has Marketing Authorization Approval in many countries including EU/EEA, Australia, Canada, and the USA. The vaccine was not available until 2014, at which time IMD due to MenB accounted for 74% of IMD disease in most European countries. This is, in part, due to vaccination programmes that targeted serogroups A, C, W, and Y. The 4CMenB vaccine is available in Greece since 2015. According to the National Immunisasion Programme is recommended only for high risk groups; however, peadiatricians are offering vaccination privetly the past three years. In this low incidence setting, the cost-effectiveness of 4CMenB vaccine use in young adults depended on it being able to generate direct and indirect protection, as seen with the MCC vaccines [6]. In a study where conjugate serogroup A, C, W, and Y (MACWY) or 4CMenB vaccine were used in UK university students, immunisation reduced overall carriage of *N*. *meningitidis*, with MenACWY affecting only the targeted capsular groups, whereas 4CMenB had a broader but modest effect regardless of capsule group [14]. More information on the impact of 4CMenB vaccine on carriage is required to support widespread use of 4CMenB and other protein based vaccines, which can be described as ‘serogroup B-substitute vaccines’, as they are used in place of vaccines that contain serogroup B polysaccharide.

In the early 21^st^ century, the proportion of MenB meningococci recovered in carriage studies ranged from 2.2% to 43.9% in Europe [3], with a study in Greece showing that MenB was predominant (34.9%) among groupable carriage isolates [14]. The aim of the present study was the characterisation of the 71 asymptomatically carried capsule group B (MenB) isolates obtained from healthy young adults by serogrouping/genogrouping, whole genome sequencing (WGS), and Meningococcal antigen Typing System (MATS) assay which is based on the serum antibody bactericidal activity rather than mucosal immunity, in order to identify the meningococcal genotypes circulating among military recruits and University students. Data on serogroup, sequence type (ST), antigen variability, Bexsero Antigen Sequence Type (BAST) and the MATS results established baseline data and indicated the possible impact of 4CMenB immunization on asymptomatic transmission in this setting.

## Material and methods

### Isolation and identification of *N*. *meningitidis*

The isolates examined were the serogroup B subset of isolates recovered from healthy young adults (military recruits or university students) enrolled in a previous carriage study [14]. All participants -in addition to the approval by the Ethics Commitee from the National School of Public Health—responded *ad hoc* to a structured self-administrated questionnaire and a written informed consent form was signed as previously described [14]. The pharyngeal swabs were immediately plated on New York City medium (OXOID LTD, Basingstoke, Hampshire, England) and incubated at 37°C in the presence of 5% CO_2_. Cultured plates were examined at 24 and 48 hours for suspected *Neisseria* colonies. Identification procedures included Gram stain, oxidase test, and a rapid carbohydrate utilization test. All *N*. *meningitidis* identified colonies were stored at -70°C in Heart Infusion Broth with 20% glycerol. In addition, the supernatant from a heat killed cell suspension was prepared and stored at -20°C using 1μl of overnight culture suspended in 200μl of PCR grade water, vortexed, heated at 100°C for 10 min, and centrifuged at 20,000 g for 12 min for further confirmation for the presence of the *porA* gene, by PCR amplification as described previously [15].

### Capsular identification

Serogroup and genogroup was determined for all isolates. Serogrouping was carried out by slide agglutination test (Remel Europe Ltd. UK) according to manufacturer’s instructions and genogrouping determined by the implementation of a multiplex PCR targeting specific capsule group genes, as described previously [16].

### Molecular characterization (MLST, PorA, FetA)

All 71 MenB isolates along with 56 of 127 meningococcal isolates belonging to a genogroup, were characterised by ‘finetyping’ (MLST and PorA and FetA typing), as described previously [17] using the PubMLST.org/neisseria database (http://pubmlst.org/neisseria/) [14]. Sequence types (ST) were defined and grouped into Clonal Complexes (ccs). PorA genotyping for variable regions 1 and 2 (VR1 and VR2) was performed as described previously [15] and compared with the variable sequences in the *Neisseria* PorA database (http://pubmlst.org/neisseria/PorA/). Similarly, the FetA variable region was also obtained for all the typable isolates, as previously described [18], and compared with variable sequences in the *Neisseria* FetA database (http://pubmlst.org/neisseria/FetA/).

Genomic DNA extraction was performed using GenElute Bacterial Genomic DNA Kit (SIGMA, Germany) following the manufacturer’s instructions. Briefly, one microliter of 18-hour culture was suspended in 180 μl of Lysis Solution T and the extraction included the optional RNase A treatment step. The eluate was stored at -20°C until sequencing. Whole genome libraries were created using Nextera XT DNA Library Preparation Kit (Illumina FC-131-1096), using Double Indexing Strategy (Nextera XT Index Kit v2 FC-131-200x) according to the manufacturer’s instructions. Each genome assembly was annotated using the pubMLST.or/neisseria sequence definition database and included the loci defining the MLST, BAST, antigen finetyping, and cgMLST v1.0 schemes.

### 4CMenB (Bexsero, GSK) vaccine antigen sequence typing (BAST)

Nucleotide sequences of *fhbp*, *nhba*, *nadA*, and *porA* variable regions 1 and 2 were obtained by WGS analysis as previously described [19]. Alleles and the corresponding protein variants were assigned using the *Neisseria* sequence definition database (http://pubmlst.org/neisseria/).

### Meningococcal antigen typing system (MATS)

To determine the proportion of strains expected to be covered by 4CMenB all isolates were analyzed by MATS ELISA. MATS ELISA was carried out at the Meningococcal Reference Laboratory, Institut Pasteur (Paris, France), one of the accredited reference laboratories that participated in the MATS standardization process [20, 21]. MATS ELISA values were calculated as antigen-specific relative potencies compared with MenB reference strains expressing each vaccine antigen [20, 22].

Predicted coverage using MATS-PBT (Positive Bactericidal Threshold) was calculated as described previously using the threshold that was established for invasive isolates [20, 21, 22]. The presence of at least one antigen with a relative potency greater than its MATS-PBT relative potency value (0.012 for fHbp, 0.294 for NHBA and 0.009 for NadA) or the presence of PorA VR2 P1.4 (the VR2 present in the OMV-NZ component of 4CMenB) was considered sufficient for an isolate to be covered by 4CMenB. Strains that did not meet these criteria were considered not covered. Estimates of the 95% confidence intervals (95% CI) for the MATS-PBTs were derived on the basis of overall assay repeatability and reproducibility (0.014–0.031 for fHbp, 0.169–0.511 for NHBA, 0.004–0.019 for NadA) [22]. These intervals defined the 95% CI of strain coverage by 4CMenB.

### Genetic diversity and association analysis

The diversity of each vaccine component was assessed using the Simpson’s index of diversity (D) [19, 23] and was calculated for each antigen using the Comparing Partitions Online Tool (http://www.comparingpartitions.info/index.php?link=Tool). A D index value near one indicated high diversity and values <1 indicated lower diversity. Cramer’s V coefficient was used to assess the association of the BAST antigenic variants with clonal complex, using SPSS version 20, with values from 0, indicating no association, to 1, indicating complete match.

## Results

### Capsular typing

Among the 180 *N*. *meningitidis* isolates recovered from 1420 pharyngeal swabs, 71 were identified as MenB either by slide agglutination test (45/71, 63.4%) or PCR (26/71; 36.6%) and included in the present study for further analysis. All 71 isolates possessed genes encoding the group B capsule (genogroup B). A complete *csb* gene (NEIS2161) sequence was assembled in 64 of the 71 (90%) isolates. In the remaining seven isolates an identifiable *csb* gene was present, although the *cps* region was incompletely assembled. Among the 26 capsule genogroup B PCR positive isolates not expressing serogroup B phenotypically, 18 (69%) presented sequence insertions or deletions switching off the gene expression (genetically phase variable ‘off’), as were four isolates identified as serogroup B by the slide agglutination test.

### Sequence typing: MLST, *porA*, *fetA*

**Fig 1:**
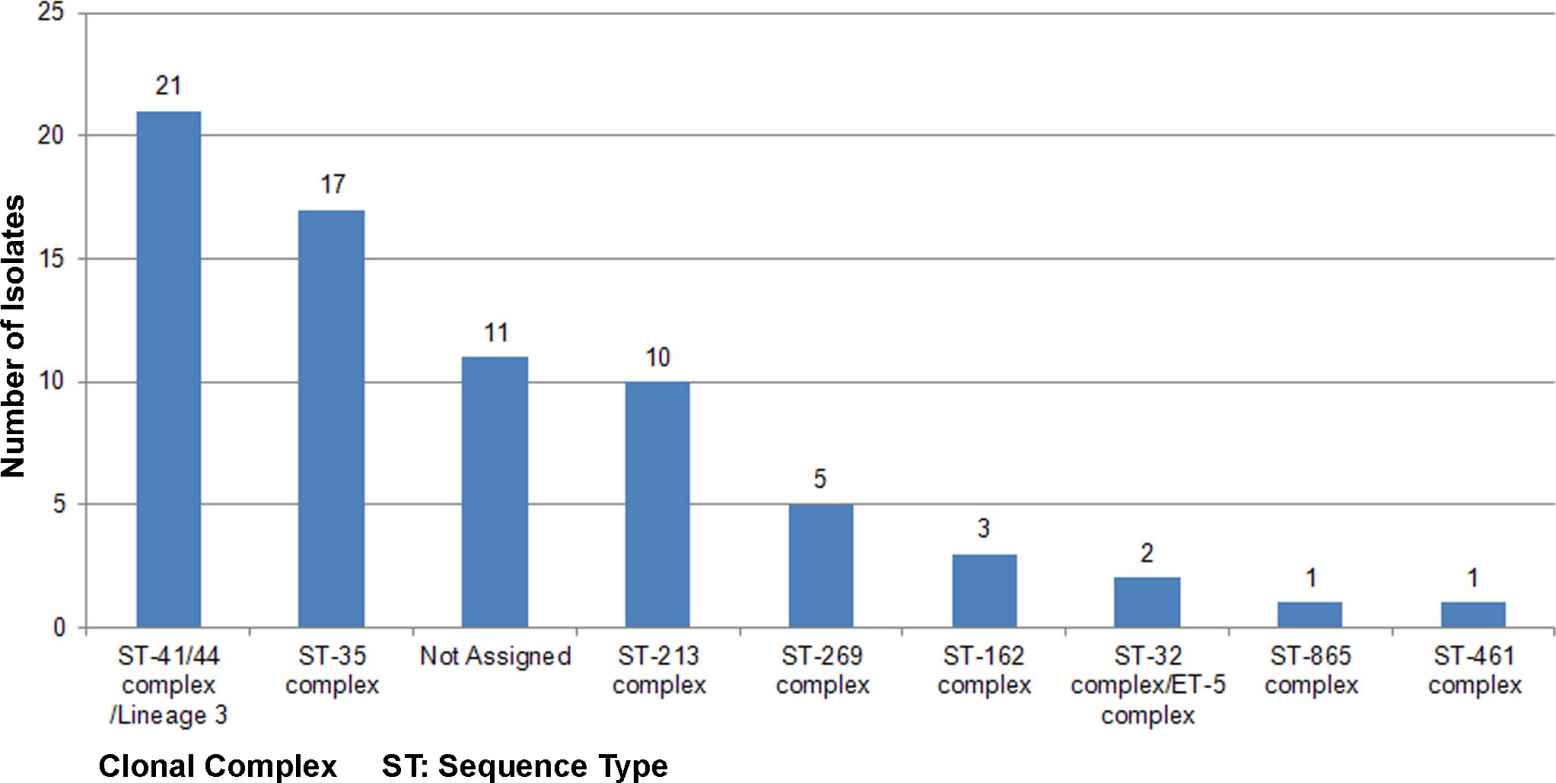
Distribution of clonal complexes (CCs) among the meningococcal group B carrier isolates.

The 71 MenB isolates were distributed into 8 distinct clonal complexes (cc). The 41/44cc was the most frequent (21/71; 30%) followed by 35cc (17/71; 23.9%) and 213cc (10/71; 14.1%). A total of 11 meningococci (15.5%) exhibited STs that were not assigned to any known cc (“Fig 1”). PorA variable region genotyping revealed variability for the two variable regions VR1 and VR2, with 36 combinations. The most frequent combinations were: P1.22–1,14 (13/71; 18.3%,); P1.22,14 (10/71; 15.4%,); P1.22,14–6 (7/71; 9.8%,); and P1.22,9 (5/71;, 7%,). The two most frequent PorA VR1 types were 22 (27/71; 38%) and 22–1 (16/71; 22.5%) while the most frequent PorA VR2 type was 14 (35.2%). The PorA variant P1.7–2,4, present in the 4CMenB vaccine, was present in seven genomes (7/71; 9.9%) and 1 (1/71; 1.4%) for VR1 and VR2 respectively (S1 Table). The *fetA* gene was present in all 71 genomes, encoding 16 different FetA variable regions. Variants F1-5 (15/71; 21.1%), F5-5 (13/71; 18.3%), and F4-1 (10/71; 14.1%) were the most frequently observed. The *fetA* gene was incompletely assembled in five isolates and in an additional isolate the gene contained a mutation introducing a stop codon and, consequently, no peptide variant was assigned in this case (“S1 Table”).

### 4CMenB molecular typing

**Fig 2:**
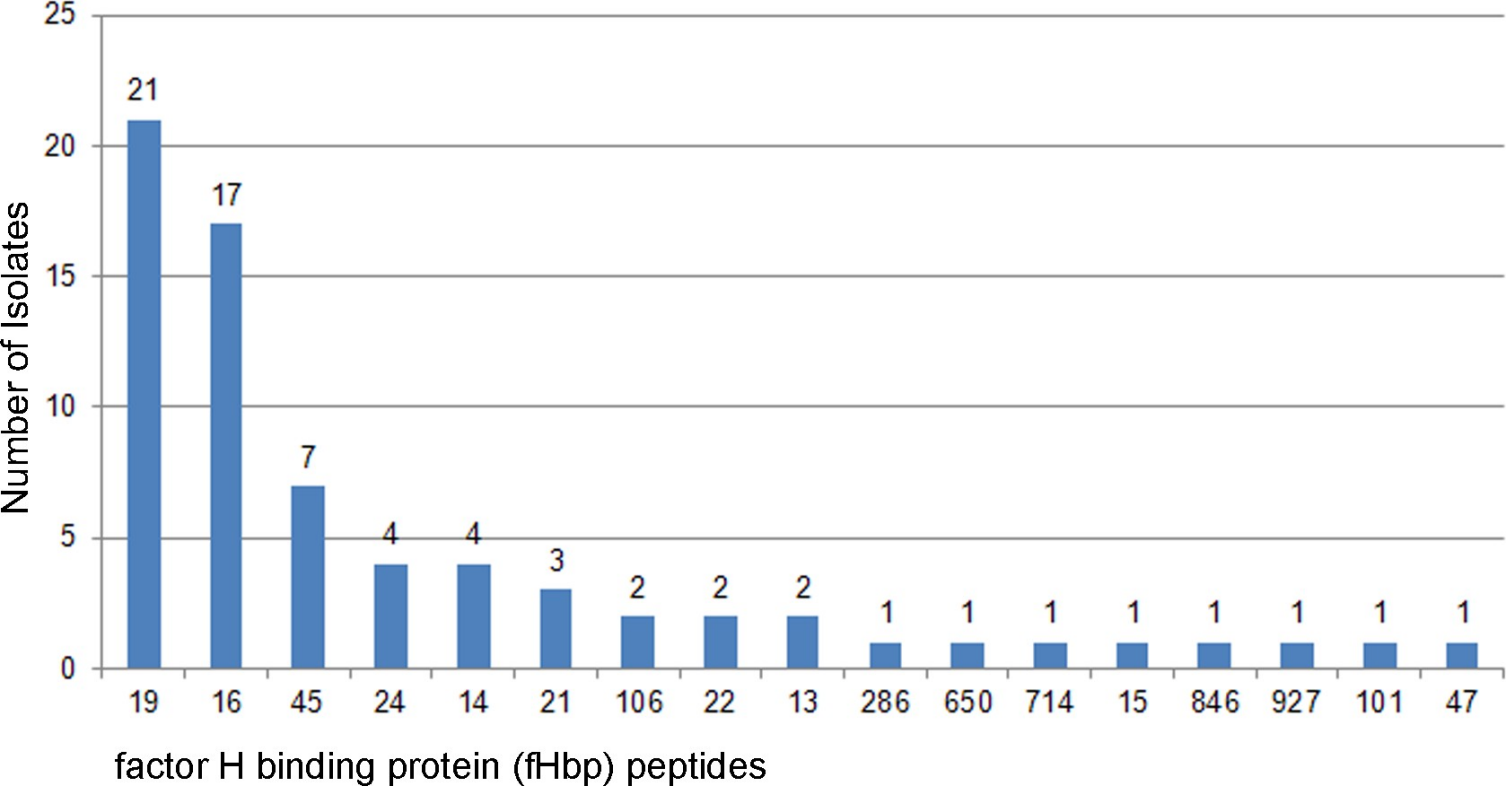
Distribution of fhbp peptides among the meningococcal group B carrier isolates.

**Fig 3:**
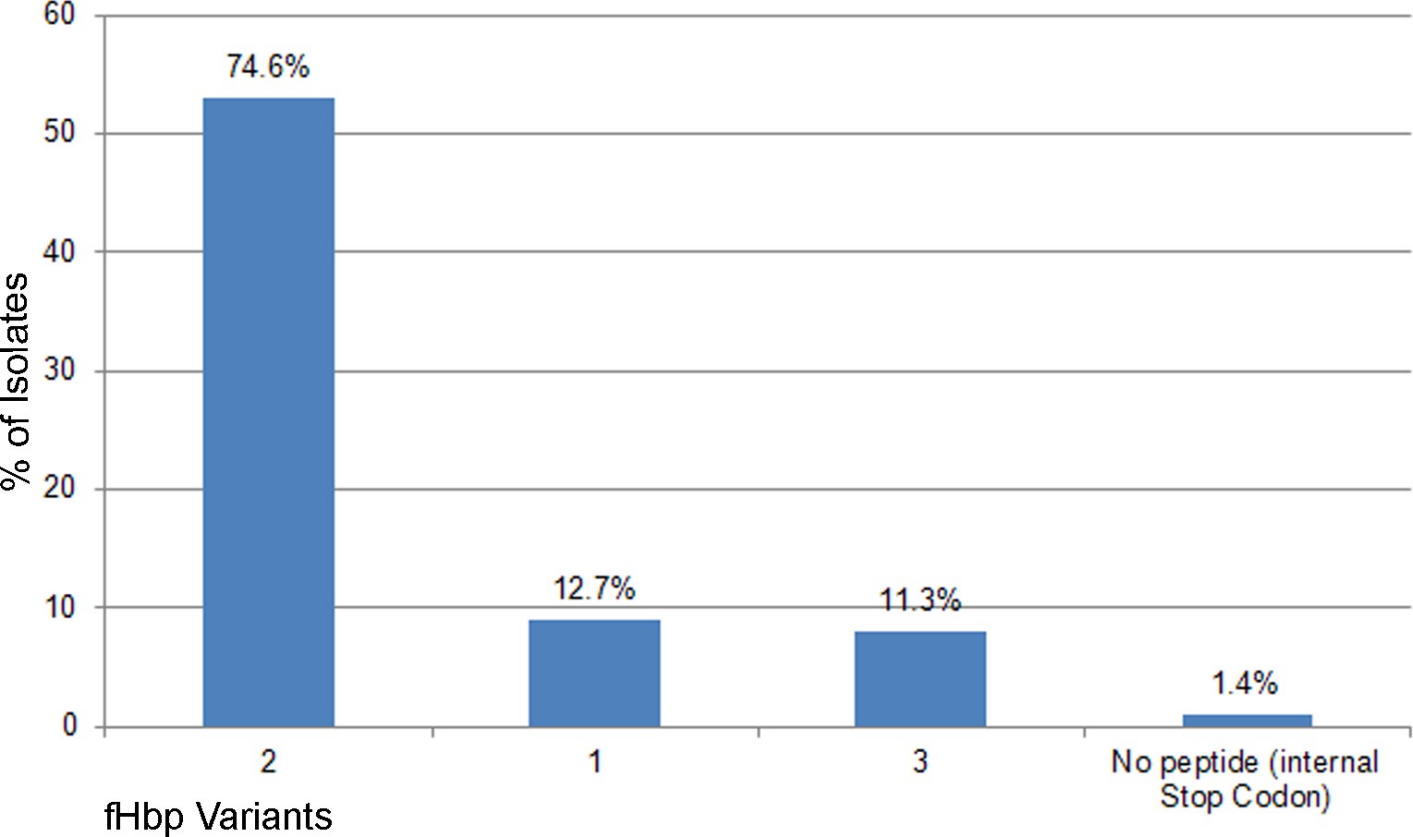
Distribution of fHbp variant peptides among the meningococcal group B carrier isolates.

**Fig 4:**
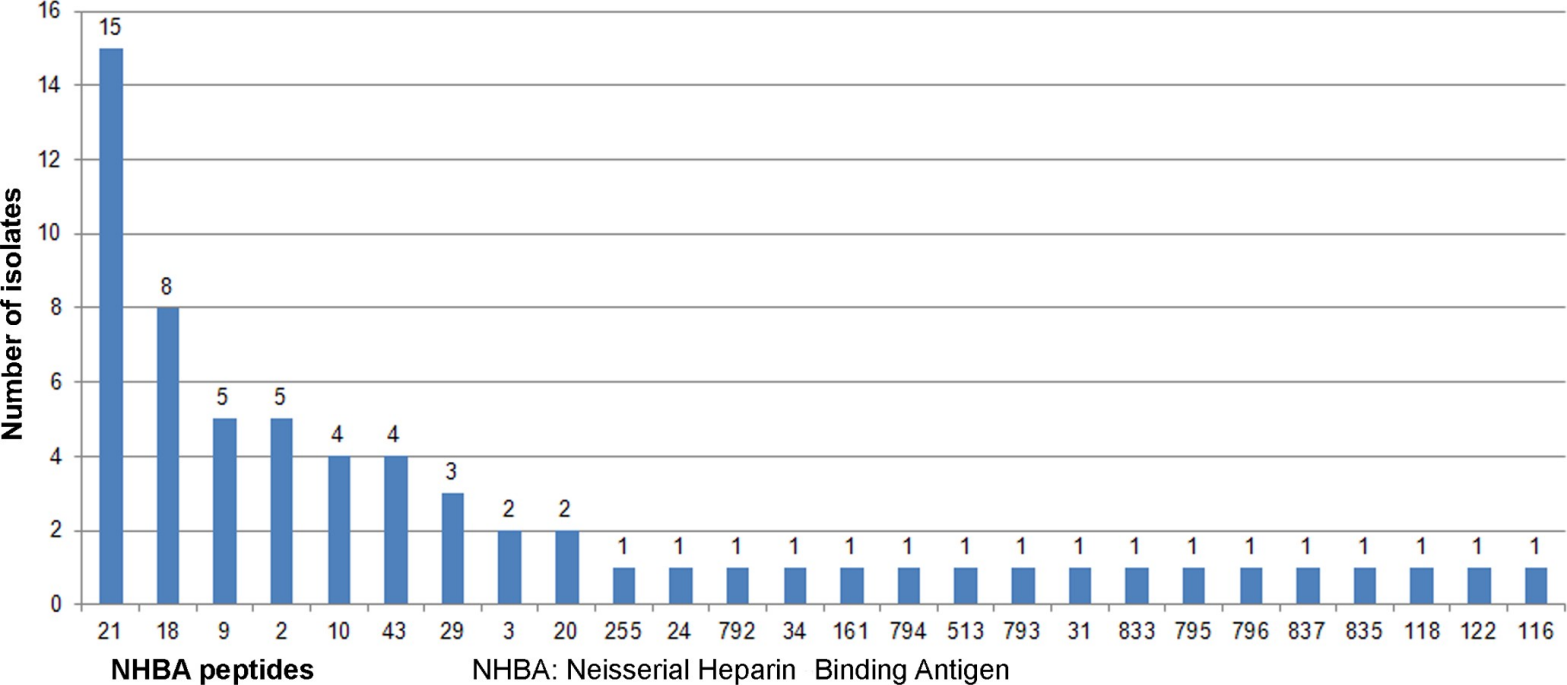
NHBA peptide distribution among the meningococcal group B carrier isolates.

The *fHbp* gene (NEIS0349), encoding the fHbp antigen, was present and was in-frame in all 71 genomes, with 17 distinct peptides encoded. The most common fHbp peptides were 19 (n = 21/71; 29,5%) and 16 (n = 17/71; 24%) (“Fig 2”). Peptide 1, which is included 4CMenB fHbp vaccine, was not found in any of the isolates examined. All three fHbp peptide variant families were present [24, 25]. Variant family 2 was observed more frequently (74.6%; 53/71), followed by variant family 1 (12.7%; 9/71) and variant family 3 (8/71; 11.2%). Among the variant family 2 fHbp peptides 19 and 16 were most frequently identified, while, peptide 45 of the family variant 3 (included in the Trumenba vaccine, Pfizer), was identified in 9.9% (7/71) of isolates. (“Fig 3”). Twenty-six different NHBA peptides were identified, of which 16 (62%) were present in a single isolate (“S1 Table”). The most frequent was peptide 21 (15/71; 21.1%), followed by peptide 18 (8/71; 11.2%), while, NHBA peptide 2, included in the 4CMenB vaccine, was observed in 4 isolates (5.6%) (“Fig 4”). The *NadA* gene (NEIS1969) was present in 12 isolates (6.9%), nine of which contained a frameshift mutation resulting in a premature stop codon. Of the three genomes with assigned peptides (1, 21, and 100), none was identified to the 4CMenB vaccine target, peptide 8 (“S1 Table”).

### Bexsero antigen sequence type (BAST)

**Fig 5:**
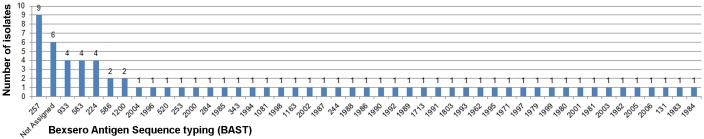
Distribution of Bexsero antigen sequence typing (BAST).

**Fig 6:**
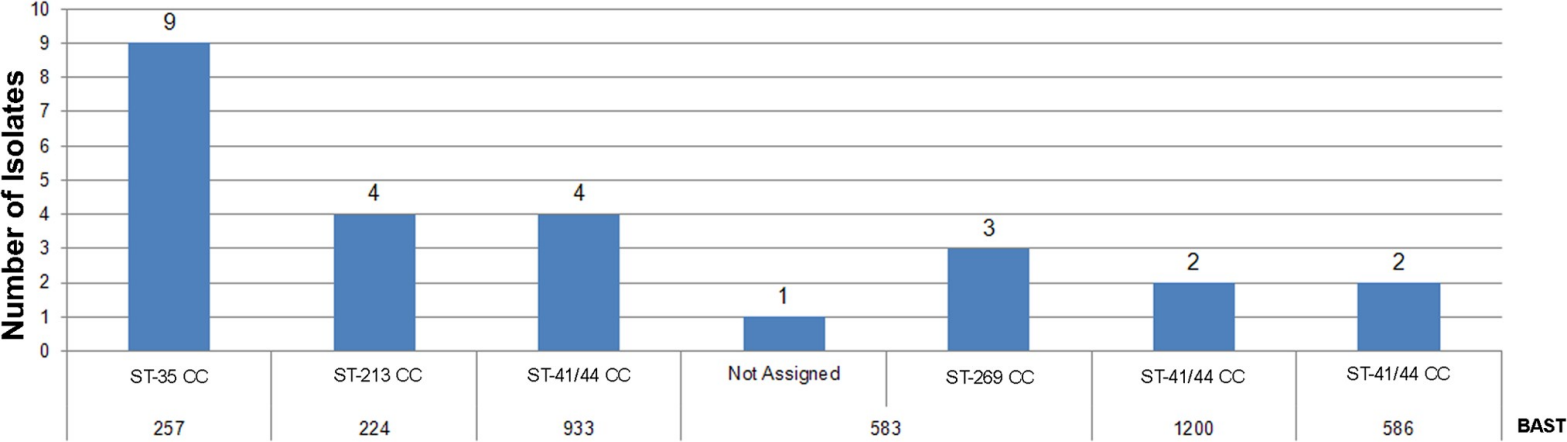
BAST association with clonal complexes as determined by MLST.

The pubMLST.org/neisseria database hosts the BAST (Bexsero Antigen Sequence Type) scheme for 4CMenB antigens and BAST types were assigned for 65 of the 71 isolates (91.5%). Forty-six (46) different BASTs were identified, 40/65 (61.5%) of which were present in only one isolate. BAST-257 was observed most frequently (9/65, 13.8%) followed by BAST-224, BAST-583, and BAST-933 (4/65, 6.1% each), representing 32.3% of the isolates (“Fig 5”). Nine BASTs were present in isolates with an ST not associated with a defined clonal complex (cc) and a 10^th^ BAST that was similarly in isolates with an ST was not assigned to a cc. The remaining BASTs, including five with incomplete profiles, were associated with eight ccs: BAST-257(n = 9) with 35cc; BAST-933 (n = 4), BAST-586 (n = 2), and BAST 1200 (n = 2) with 41/44cc; BAST-224 (n = 4) with 213cc; and BAST-583 (n = 3) with 269cc (“Fig 6”).

### Meningococcal antigen typing system (MATS) phenotype

The MATS analysis for 70 of the 71 MenB isolates predicted coverage by at least one antigen (fHbp, NHBA, NadA, or PorA) for 38% (27/70) of the isolates, with the NHBA antigen providing the highest contribution (16/70; 22.9%). Cross protective fHbp variants were present in 5 isolates (5/70; 7.1%), with in 4 in combination with NHBA (4/70; 5.7%). NadA coverage was detected in 1 isolate (1/70; 1.4%), while PorA (1/70, 1.4%) was found in combination with NHBA (“Table 1”).

**Table 1:** Contribution of each antigen and of combined antigens to coverage, predicted by MATS^a^.

Antigen Combination	Number of isolates (%)	coverage of each antigen combination (%)	% coverage of combined antigen group
**No antigen**	43 (61.4)	0	0
**fHbp**^b^	5 (7.1)	7.1%	31.4%
**NHBA**^c^	16 (22.9)	22.9%	
**NadA**^d^	1 (1.4)	1.4%	
**PorA**^e^**+NHBA**	1 (1.4)	1.4%	7.1%
**fHbp +NHBA**	4 (5.7)	5.7%	

*^a^MATS*:Meningococcal Antigen Typing System,

*^b^fHbp*:factor H binding protein,

*^c^NHBA*: Neisserial Heparin Binding Protein,

^d^*NadA*:Neisserial Adhesion A,

^e^*PorA*: outer membrane Protein A

fHbp-1 sub-variants were found in all 9 isolates showing detectable expression levels of the fHbp antigen (alone or with the NHBA combination) while 1 of the 3 isolates expressing the NadA peptide 1 had an RP value above the detection threshold for this specific antigen. Simpson’s index of diversity (D) indicated that NHBA was the most diverse antigen (0.926 [0.892–0.959 CI 95%]), followed by PorA VR2 (0.847 [0.773–0.922 CI 95%]), fHbp (0.845 [0.79–0.9 CI 95%]), PorA VR1 (0.796 [0.727–0.866 CI 95%]), and NadA (0.083 [1.000–0.173]). Using the Cramer’s V coefficient calculation an association was observed between BAST-257 and clonal complex ST-35.

## Discussion

At the time of writing IMD, particularly that caused by serogroup B meningococci, was incompletely controlled by immunisation, largely because of the antigenic and genetic diversity of *Neisseria meningitidis*. In particular, there were uncertainties as to the ability of the protein-based vaccines to generate herd protection (immunity), especially given the diversity of meningococci meningococci isolated from asymptomatic carriers [1].

Previous studies have shown that the introduction of monovalent meningococcal group C polysaccharide conjugate vaccine (MCC) in national immunization programs reduced MenC IMD through herd protection, by reducing the transmission of the epidemic strain among asymptomatic carriers [11,12]. Similarly, the national introduction of a strain-specific outer membrane vesicle vaccine (MeNZB) in New Zealand reduced IMD cases from the epidemic clone, with limited evidence for cross-protection [26] with a potential impact on the acquisition of carriage of the epidemic strain [27]. Similar impact on the acquisition of carriage of an epidemic isolate was also suggested for another OMV vaccine (MenBVac) [28]. The impact of meningococcal vaccines on asymptomatic transmission is an important aspect in the evaluation of the impact of new meningococcal vaccines on disease, particularly their cost-effectiveness [29]. Recently, a multicomponent protein-based vaccine (4CMenB, Bexsero), designed to target MenB disease in the absence of a vaccine against the group B capsule, was licensed in many European countries including Greece; however, a carriage study in UK has shown that the potential broad additional effect on commensal *Neisseria* and non-disesae associated *N*. *meningitidis* cannot be predicted [14]. While no effect on carriage was observed 1 month after completion of the vaccine course, this effect was from 3 months after dose two of 4CMenB. This effect was observed for all meningococci but not specifically of serogroup B isolates. This global effect could be expected as this vaccine does not target the capsule but surface exposed proteins that can be shared by the isolates regardless the serogroup. However, an effect on serogroup B should be analysed on the basis of the expression data (e.g. MATS as performed in our study) but that still require a “correlate of protection” for carriage isolates.

No correlate of protection against acquisition of carriage at the mucosal surfaces was available to this study, and this prevented this issue being addressed directly, by assaying the mucosal immunity against meningococcal isolates; however, data from animal models have demonstrated the detection of immune response in the mucosal secretions for protein-based vaccines, which correlated with the prevention against intranasal colonization by isolates showing multiple matching with vaccine antigen [28,30]. Our data clearly show that the expression of antigens of the 4CMenB vaccine in carriage isolates as suggested by the MATS data.

Here, the 4CMenB vaccine antigens were assessed in a collection of MenB isolates obtained in Greece from asymptomatic carriage in young adults aged 17–26 years. The study included isolates obtained immediately before and after the introduction of the 4CMenB vaccine in Greece; however, this initial implementation in Greece was unlikely to have affected carriage in the age group sampled. Consequently, this study represented a pre-vaccine meningococcal carriage sample in an age group with high carriage [14, 31]. It provides a reference sample for that will be essential for: (i) assessing the potential impact of the 4CMenB vaccine on carriage; (ii) monitoring the effect of vaccine use on antigen evolution among carried meningococci; and (iii) analyzing any genomic changes in the meningococcal population circulating in Greece. The application of WGS, in combination with the MATS assay, provided a combination of genotypic and phenotypic characterisation for these isolates. However, a correlate of protection against acquisition of carriage is still lacking to evaluate the WGS/MATS data from carriage isolates.

Among the isolates that possessed a *cps* region encoding the group B capsule (genogroup B, MenB), over a third (n = 26, 36.6%) were defined as phenotypically non-groupable (NG) by agglutination, suggesting down-regulation of capsule expression during carriage. This is consistent with many previous studies including a recent investigation in Italy [10], where 40% of group B carrier isolates were NG. A total of 21 (81%) of the NG isolates exhibited intact capsule operons, with 18 (69%) containing a *csb* gene predicted to be phase variable off. This demonstrates the importance of using WGS data to investigate such isolates, as this is the most practical and cost-effective means of determining the presence and expression status of the capsule operon. The majority of genogroup B isolates analysed in this study were represented by clonal complexes 41/44cc, 35cc, 32cc, 213cc, 269cc, and 162cc. This was also consistent with previous observations from a variety of high-income countries [10, 32]. As the relationship between asymptomatic carriage and the development of invasive disease remains incompletely understood, it is important to collect isolates belonging to hyperinvasive genotypes worldwide.

The peptide sequences of the principal 4CMenB vaccine antigens, summarized by the BAST type, were extracted from the assembled WGS data using the tools integrated into the https://pubmlst.org/neisseria/ [19]. As has been reported previously for disease isolates [19, 33], each of the vaccine antigens exhibited extensive sequence variation among the 71 genogroup B isolates, with NHBA being the most diverse antigen (“Table 1”). The strong correlation of cc with both BAST and individual antigen variant, such that in the absence of antigen data cc could be used as a surrogate for likely cross protection, was consistent with several studies of invasive disease isolates [19, 33, 34]. Similarly consistent with studies of IMD isolates, exact matches to the vaccine antigen variants were rare: PorA VR2 (P1.4) was found in only one isolate belonging to 41/44 cc; NHBA peptide 2 was found in five isolates (7.6%); the fHbp target peptide 1 and NadA target peptide 8 were not present in this collection of isolates. The prevalence of PorA VR2 P1.4 (1, 1.4%) was considerably lower to that was found among invasive isolates (7%) in Greece [35]. Consequently, any impact of the 4CMenB vaccine on carriage isolates will depend on immunological cross protection of the type assessed in the MATS assay.

All three fHbp variant families were present among the 71 carriage isolates. Consistent with the findings of other carriage studies, variant family 2 was more abundant than variant family 1 [10, 32]. No cross reactivity of the 4CMenB vaccine antibodies with isolates containing variant family 2 or 3 peptides, was observed (“S1 Table”); however, 90% of the isolates containing fHbp variant family 1 peptides were MATS RP positive (“S1 Table”). Similar data indicating the expression of fHbp family 1 among 95% of carriage isolates harbouring this variant were reported from France using ELISA [36]. There were 26 different peptides for NHBA, and only 5 isolates (7.7%) contained the 4CMenB vaccine variant, peptide 2. The presence of the NHBA peptide 2 among group B meningococci from carriers is 30%, lower than the presence of this peptide among IMD group B isolates in Greece (10.1%) [35]. This is in contrast with a recent carriage study from Spain, which found comparable levels of NHBA peptide 2 among carriers and IMD associated group B isolates of 3% and 4% for carrier and invasive group B isolates respectively [32].

While BAST data demonstrated a high diversity of the fHbp, NHBA, NadA, and PorA antigens, this was mainly due to fHbp and NHBA, and a limited number of BASTs occurred at high frequency [37]. BAST-1 (fHbp-1, NHBA-2, NadA-8, PorA P1.7–2,4), corresponding to the 4CMenB vaccine components, was absent from this dataset which was consistent with the fact that the vaccine formulation was assembled from meningococci from multiple ccs. MATS estimated the overall potential 4CMenB vaccine coverage at 38.6%, which was substantially lower than the estimated coverage for MenB IMD isolates (89.2%) from Greece for 1999–2010 [35]. A total of eight isolates (11.4%) predicted to be covered by MATS contained capsule group B-encoding *cps* regions but were non-groupable (NG) by serological methods. One limitation of this study was that no correlation had been established between the level of antibodies needed for protection at the mucosal level and the levels of expression of the antigens targeted by the 4CMenB.

In conclusion, this study demonstrates the utility of WGS data in the characterisation of meningococcal carriage isolates to assess the prevalence of vaccine antigens, including the presence and expression of polysaccharide and protein antigens. The BAST antigens found among carried isolates were highly diverse, which was reflected by a low level of predicted cross-protection of 4CMenB against carried MenB isolates. As the majority of carriage MenB isolates do not belong to hyperinvasive linages, this observation does not necessarily preclude the use of 4CMenB to generate herd immunity against IMD. To affect disease rates, it will be important to disrupt the transmission of hyperinvasive meningococci; indeed, it may well be preferable that the transmission of less invasive variants of any meningococcal group is not impacted by immunisation. At the time of writing, a number of large-scale vaccination and carriage investigations were underway and the data generated from these, using approaches similar to those reported here, will provide a definitive answer as to the impact of this vaccine on meningococcal transmission.

## Supporting information

S1 Table Molecular characterization, capsular typing, 4CMenB vaccine molecular typing and MATS of the meningococcal group B carriage isolates included in the study. (DOC)
