# Supplementary material for: Characterization of meningococcal carriage isolates from Greece by whole genome sequencing: Implications for 4CMenB vaccine implementation
Source: PLoS One. 2018 Dec 28;13(12):e0209919. doi: 10.1371/journal.pone.0209919 (PMC6310245; doi:10.1371/journal.pone.0209919)
Supplement: S1 Table — (DOC) [file pone.0209919.s001.doc]

**S1 Table: Molecular characterization , capsular typing, 4CMenB vaccine molecular typing and MATS of the Meningococcal group B carriage isolates included in the study**

**Poly**= polyvalent, **NG**= non-groupable, **NA**= Not Assigned, **FS**= gene with a frameshift mutation, **FS-PVOFF**= gene with a frameshift mutation resulting phase variable off, **ND**= Not determined, **BAST**=Bexsero Antigen Sequence Typing scheme, **MATS**= **M**eningococcal **A**ntigen **T**yping **S**ystem. Strains showing a RP for the antigen >PBT were defined as “+’’ and those with a RP <PBT were defined as “-‘’ following the order *fH*bp, nhba , nadA and PorA=1.4 (included in the vaccine) defined as + or a different PorA defined as “-“.

| **isolate** | **porA  PCR** | **Genogroup  by PCR** | **Serogroup  slide agglutination** | **capsule  group  WGS** | **PorA  VR1** | **PorA  VR2** | **FetA  VR** | **ST** | **clonal  complex** | **fHbp peptide** | **fHbp  variant familly** | **NHBA  peptide** | **nadA  presence** | **NadA Variant** | **NadA  peptide** | **BAST** | **MATS** |
| --- | --- | --- | --- | --- | --- | --- | --- | --- | --- | --- | --- | --- | --- | --- | --- | --- | --- |
| 1 | + | B | Poly | B | 21 | 16 | F3-6 | 11535 | NA | 13 | 1 | 833 | - |  | 0 | 1962 | +, -, -, - |
| 2 | + | B | B | B | 22 | 14-6 | F1-5 | 191 | ST-41/44 | 19 | 2 | ND | - |  | 0 | ND | -, -, -, - |
| 3 | + | B | B | B | 7-2 | 4 | F1-5 | 41 | ST-41/44 | 19 | 2 | 2 | - |  | 0 | 284 | -, +, -, + |
| 4 | + | B | B | B | 22 | 14 | F5-9 | 162 | ST-162 | 927 | 2 | ND | - |  | 0 | ND | -, +, -, - |
| 5 | + | B | NG | B | 22-1 | 14 | F4-1 | 35 | ST-35 | 16 | 2 | 21 | - |  | 0 | 257 | -, -, -, - |
| 6 | + | B | Poly | B | 22-1 | 14 | F4-1 | 160 | ST-35 | 16 | 2 | 21 | - |  | 0 | 257 | -, -, -, - |
| 7 | + | B | B | B | 22-1 | 14-50 | F4-1 | 160 | ST-35 | 16 | 2 | 835 | - |  | 0 | 1971 | -, -, -, - |
| **isolate** | **porA  PCR** | **Genogroup  by PCR** | **Serogroup  slide agglutination** | **capsule  group  WGS** | **PorA  VR1** | **PorA  VR2** | **FetA  VR** | **ST** | **clonal  complex** | **fHbp peptide** | **fHbp  variant familly** | **NHBA  peptide** | **nadA  presence** | **NadA Variant** | **NadA  peptide** | **BAST** | **MATS** |
| 8 | + | B | NG | B | 22-1 | 14 | ND | 160 | ST-35 | 16 | 2 | 21 | - |  | 0 | 257 | -, -, -, - |
| 9 | + | B | B | B | 22 | 14-51 | F5-9 | 162 | ST-162 | 21 | 2 | 20 | - |  | 0 | 1979 | -, +, -, - |
| 10 | + | B | B | B | 17 | 16-28 | F5-5 | 136 | ST-41/44 | 24 | 2 | 10 | - |  | 0 | 1200 | -, +, -, - |
| 11 | + | B | B | B | 22 | 14-6 | F1-5 | 3919 | ST-41/44 | 19 | 2 | 43 | - |  | 0 | 933 | -, -, -, - |
| 12 | + | B | B | B | 17-1 | 23-2 | F1-5 | 1097 | ST-41/44 | 19 | 2 | 31 | - |  | 0 | 343 | -, -, -, - |
| 13 | + | B | B | B | 22 | 14-6 | F1-5 | 11625 | ST-41/44 | 19 | 2 | 43 | - |  | 0 | 933 | -, -, -, - |
| 14 | + | B | NG | B | 22 | 14 | F1-5 | 11626 | NA | 106 | 2 | ND | - |  | 0 | ND | -, -, -, - |
| 15 | + | B | B | B | 22-1 | 14 | F4-1 | 160 | ST-35 | 16 | 2 | 21 | - |  | 0 | 257 | -, -, -, - |
| 16 | + | B | NG | FS-PV OFF | 22-30 | 14 | F5-5 | 11537 | ST-213 | 22 | 2 | 18 | + | NadA-4/5 | FS | 1980 | -, -, -, - |
| 17 | + | B | NG | B | 22 | 16-118 | F3-3 | 11538 | ST-41/44 | 24 | 2 | 10 | - |  | 0 | 1981 | -, +, -, - |
| 18 | + | B | B | B | 22 | 14 | F5-5 | 213 | ST-213 | 45 | 3 | 18 | + | NadA-4/5 | FS | 224 | -, -, -, - |
| 19 | + | B | B | B | 22-1 | 4-40 | F5-19 | 160 | ST-35 | 16 | 2 | 21 | - |  | 0 | 1982 | -, -, -, - |
| **isolate** | **porA  PCR** | **Genogroup  by PCR** | **Serogroup  slide agglutination** | **capsule  group  WGS** | **PorA  VR1** | **PorA  VR2** | **FetA  VR** | **ST** | **clonal  complex** | **fHbp peptide** | **fHbp  variant familly** | **NHBA  peptide** | **nadA  presence** | **NadA Variant** | **NadA  peptide** | **BAST** | **MATS** |
| 20 | + | B | B | B | 12-1 | 13-1 | F1-66 | 11539 | NA | 14 | 1 | 24 | - |  | 0 | 1803 | +, -, -, - |
| 21 | + | B | B | B | 18 | 25-11 | F5-5 | 5985 | ST-269 | 15 | 1 | 21 | - |  | 0 | 1983 | +, +, -, - |
| 22 | + | B | B | B | 22 | 14-49 | F5-5 | 213 | ST-213 | 45 | 3 | ND | + | NadA-4/5 | FS | ND | -, -, -, - |
| 23 | + | B | Poly | B | 22 | 14 | F5-5 | 213 | ST-213 | 45 | 3 | ND | + | NadA-4/5 | FS | ND | -, -, -, - |
| 24 | + | B | B | B | 22 | 9 | F1-5 | 11508 | ST-269 | 19 | 2 | 9 | - |  | 0 | 583 | -, -, -, - |
| 25 | + | B | B | B | 22 | 14 | F5-5 | 213 | ST-213 | 45 | 3 | 18 | + | NadA-4/5 | FS | 224 | -, -, -, - |
| 26 | + | B | NG | FS-PVOFF | 22 | 9 | F5-12 | 10922 | ST-269 | 19 | 2 | ND | - |  | 0 | ND | -, -, -, - |
| 27 | + | B | B | B | 22 | 9 | F1-5 | 11540 | NA | 19 | 2 | 9 | - |  | 0 | 583 | -, -, -, - |
| 28 | + | B | B | B | 7-2 | 30-3 | F1-66 | 1111 | NA | 14 | 1 | 837 | - |  | 0 | 1984 | +, +, -, - |
| 29 | + | B | B | B | 7-2 | 14 | F1-7 | 1947 | ST-41/44 | 16 | 2 | 161 | - |  | - | 1985 | -, +, -, - |
| 30 | + | B | B | B | 7-1 | 1-1 | F4-1 | 11502 | ST-41/44 | 19 | 2 | 29 | - |  | - | 586 | -, +, -, - |
| 31 | + | B | B | B | 7-1 | 1-1 | F4-1 | 11502 | ST-41/44 | 19 | 2 | 29 | - |  | - | 586 | -, -, -, - |
| **isolate** | **porA  PCR** | **Genogroup  by PCR** | **Serogroup  slide agglutination** | **capsule  group  WGS** | **PorA  VR1** | **PorA  VR2** | **FetA  VR** | **ST** | **clonal  complex** | **fHbp peptide** | **fHbp  variant familly** | **NHBA  peptide** | **nadA  presence** | **NadA Variant** | **NadA  peptide** | **BAST** | **MATS** |
| 32 | + | B | Poly | B | 18 | 25 | F1-84 | 414 | ST-41/44 | 19 | 2 | 2 | - |  | - | 1986 | -, +, -, - |
| 33 | + | B | B | B | 18-7 | 9-5 | F3-9 | 877 | ST-41/44 | 19 | 2 | 792 | - |  | - | 1987 | -, +, -, - |
| 34 | + | B | B | B | 22-1 | 14 | F5-2 | 1976 | NA | 14 | 1 | 2 | + | NadA-4/5 | 21 | 131 | +, +, -, - |
| 35 | + | B | Poly | B | 22-1 | 14 | F3-6 | 160 | ST-35 | 16 | 2 | 21 | - |  | - | 257 | -, -, -, - |
| 36 | + | B | B | B | 22-1 | 14 | F4-1 | 160 | ST-35 | 16 | 2 | 21 | - |  | - | 257 | -, -, -, - |
| 37 | + | B | B | B | 17 | 9 | F1-7 | 163 | NA | 19 | 2 | 793 | - |  | - | 1988 | -, -, -, - |
| 38 | + | B | B | B | 22 | 14-6 | F1-7 | 44 | ST-41/44 | 19 | 2 | 29 | - |  | - | 1163 | -, -, -, - |
| 39 | + | B | B | B | 19 | 15 | F5-1 | 34 | ST-32 | 0 | 0 | 3 | + | NadA-1 | 1 | 2006 | -, -, +, - |
| 40 | + | B | NG | B | 22 | 14 | F5-5 | 213 | ST-213 | 22 | 2 | 18 | + | NadA-4/5 | FS | 1989 | -, -, -, - |
| 41 | + | B | Poly | B | 12-1 | 23 | F1-5 | 11506 | NA | 19 | 2 | 34 | - |  | - | 1990 | -, -, -, - |
| 42 | + | B | B | B | 7-4 | 1 | F1-114 | 11507 | ST-41/44 | 846 | 2 | 794 | - |  | - | 1991 | -, -, -, - |
| **isolate** | **porA  PCR** | **Genogroup  by PCR** | **Serogroup  slide agglutination** | **capsule  group  WGS** | **PorA  VR1** | **PorA  VR2** | **FetA  VR** | **ST** | **clonal  complex** | **fHbp peptide** | **fHbp  variant familly** | **NHBA  peptide** | **nadA  presence** | **NadA Variant** | **NadA  peptide** | **BAST** | **MATS** |
| 43 | + | B | B | B | 22 | 9 | F1-5 | 11508 | ST-269 | 19 | 2 | 9 | - |  | - | 583 | NA, NA, NA, - |
| 44 | + | B | Poly | B | 22-1 | 14 | F4-1 | 11509 | ST-35 | 16 | 2 | 21 | - |  | - | 257 | -, +, -, - |
| 45 | + | B | B | B | 7-4 | 1-12 | F3-6 | 11510 | NA | 286 | 2 | 9 | - |  | - | 1992 | -, -, -, - |
| 46 | + | B | B | B | 22 | 9 | F1-5 | 11508 | ST-269 | 19 | 2 | 9 | - |  | - | 583 | -, -, -, - |
| 47 | + | B | NG | FS | 22 | 14-6 | F5-1 | 336 | NA | 106 | 2 | 122 | - |  | - | 1993 | -, -, -, - |
| 48 | + | B | B | B | 22 | 14-6 | F1-5 | 207 | ST-41/44 | 19 | 2 | 43 | - |  | - | 933 | -, -, -, - |
| 49 | + | B | Poly | B | 22 | 14 | F5-9 | 162 | ST-162 | 21 | 2 | 20 | - |  | - | 244 | -, +, -, - |
| 50 | + | B | Poly | B | 7-2 | 13-1 | ND | 160 | ST-35 complex | 16 | 2 | 21 | - |  | - | 1994 | -, -, -, - |
| 51 | + | B | B | B | 22 | 14-6 | F1-5 | 191 | ST-41/44 | 19 | 2 | 43 | - |  | - | 933 | -, -, -, - |
| 52 | + | B | Poly | B | 18 | 25 | F1-7 | 3447 | ST-41/44 | 14 | 1 | 2 | - |  | - | 1081 | +, +, -, - |
| 53 | + | B | B | B | 7-2 | 30-8 | F5-2 | 3645 | ST-865 | 714 | 1 | 255 | - |  | - | 1995 | +, -, -, - |
| 54 | + | B | B | B | 17 | 16-28 | F5-5 | 136 | ST-41/44 | 24 | 2 | 10 | - |  | - | 1200 | -, +, -, - |
| 55 | + | B | Poly | B | 22-1 | 14 | F4-1 | 160 | ST-35 | 16 | 2 | 21 | - |  | - | 257 | -, -, -, - |
| **isolate** | **porA  PCR** | **Genogroup  by PCR** | **Serogroup  slide agglutination** | **capsule  group  WGS** | **PorA  VR1** | **PorA  VR2** | **FetA  VR** | **ST** | **clonal  complex** | **fHbp peptide** | **fHbp  variant familly** | **NHBA  peptide** | **nadA  presence** | **NadA Variant** | **NadA  peptide** | **BAST** | **MATS** |
| 56 | + | B | Poly | B | 22 | 14 | F3-9 | 11503 | ST-213 | 16 | 2 | 18 | + | NadA-4/5 | FS | 1996 | -, -, -, - |
| 57 | + | B | B | B | 22 | 14 | F5-5 | 213 | ST-213 | 45 | 3 | 18 | + | NadA-4/5 | FS | 224 | -, -, -, - |
| 58 | + | B | Poly | B | 7-2 | 16 | F3-3 | 5331 | ST-32 | 13 | 1 | 3 | + | NadA-1 | 100 | 1997 | +, -, -, - |
| 59 | + | B | B | B | 17 | 16-3 | F5-5 | 136 | ST-41/44 | 24 | 2 | 10 | - |  | - | 253 | -, +, -, - |
| 60 | + | B | Poly | B | 22-1 | 14 | F4-1 | 160 | ST-35 | 650 | 1 | 21 | - |  | - | 1713 | +, -, -, - |
| 61 | + | B | Poly | B | 22 | 14-15 | F5-5 | 213 | ST-213 | 45 | 3 | 18 | + | NadA-4/5 | FS | 1998 | -, -, -, - |
| 62 | + | B | poly | other | 21 | 26-2 | ND | 35 | ST-35 | 101 | 2 | 21 | - |  | - | 1999 | -, -, -, - |
| 63 | + | B | B | B | 22 | 14 | F3-9 | 11512 | NA | 21 | 2 | 2 | - |  | - | 2000 | -, +, -, - |
| 64 | + | B | B | B | 7-2 | 9 | F1-5 | 1489 | ST-41/44 | 19 | 2 | 116 | - |  | - | 2001 | -, +, -, - |
| 65 | + | B | NG | B | 22-1 | 14 | ND | 160 | ST-35 | 16 | 2 | 21 | - |  | - | 257 | -, -, -, - |
| 66 | + | B | B | B | 18-7 | 9 | F5-8 | 11513 | ST-41/44 | 19 | 2 | 795 | - |  | - | 2002 | -, -, -, - |
| 67 | + | B | B | B | 19-2 | 13-2 | F3-9 | 1946 | ST-461 | 47 | 3 | 118 | - |  | - | 520 | -, +, -, - |
| 68 | + | B | B | B | 22-1 | 16-8 | F1-5 | 11514 | ST-35 | 16 | 2 | 21 | - |  | - | 2003 | -, +, -, - |
| **isolate** | **porA  PCR** | **Genogroup  by PCR** | **Serogroup  slide agglutination** | **capsule  group  WGS** | **PorA  VR1** | **PorA  VR2** | **FetA  VR** | **ST** | **clonal  complex** | **fHbp peptide** | **fHbp  variant familly** | **NHBA  peptide** | **nadA  presence** | **NadA Variant** | **NadA  peptide** | **BAST** | **MATS** |
| 69 | + | B | B | B | 22 | 14 | F5-5 | 213 | ST-213 | 45 | 3 | 18 | + | NadA-4/5 | FS | 224 | -, -, -, - |
| 70 | + | B | NG | B | 22-1 | 14 | ND | 11504 | ST-35 | 16 | 2 | 513 | - |  | - | 2004 | -, -, -, - |
| 71 | + | B | B | B | 22-1 | 14 | ND | 160 | ST-35 | 16 | 2 | 796 | - |  | - | 2005 | -, -, -, - |
